# The Impact of Successful Transurethral Indwelling Catheter Removal on Health-Related Quality of Life in Patients Undergoing Neurological Rehabilitation

**DOI:** 10.3390/neurolint18010012

**Published:** 2026-01-06

**Authors:** Anke K. Jaekel, Manuel Pickermann, Ann Katrin Walter, Anna-Lena Butscher, John Bitter, Franziska I. Winterhagen, Ruth Kirschner-Hermanns, Stephanie C. Knüpfer

**Affiliations:** 1 Department of Neuro-Urology, Clinic for Urology, University Hospital Bonn, 53127 Bonn, Germany; 2 Department of Neuro-Urology, Johanniter Neurological Rehabilitation Center Godeshoehe GmbH, 53177 Bonn, Germany

**Keywords:** bladder, urinary incontinence, neurology, rehabilitation, indwelling catheter, urinary catheter, quality of life

## Abstract

Background/Objectives: Patients with acute severe neurological disorders often receive a transurethral indwelling catheter (TUIC) during their initial treatment. These TUICs often remain in place until the transfer to a rehabilitation or a long-term care facility. There are no systematic concepts for bladder management and no data regarding the impact on the catheter associated, health-related quality of life (HRQoL) in this patient group. The aim of this study was to investigate the impact of successful TUIC removal on the HRQoL of those affected and to contribute to the development of systematic bladder management. Methods: A prospective longitudinal study was conducted on 33 patients treated at a neurological rehabilitation centre due to acute severe neurological disorders. The HRQoL was assessed using the SF-36 Health Survey prior to and following the TUIC removal. The influence of urinary incontinence was analysed. The mean differences were determined using a one-sample *t*-test adjusted for age and gender. Results: TUIC removal was successful in 61.8% (21/33). The SF-36 Health Survey showed the following improvements (adj. mean diff., 95% CI, *p*-value): Mental Component Summary measure (4.36, 0.34; 8.38, *p* = 0.035), Role-Emotional (20.89, 0.54; 41.24, *p* = 0.045), Physical Functioning (10.03, 3.18; 16.88, *p* = 0.007). The comparison between incontinent and continent patients showed a poorer HRQoL for the incontinent group. Conclusions: Successful TUIC removal has a positive influence on psychological/emotional aspects and physical functioning. Structured bladder management that considers the physical and psychological aspects of patients and nursing staff, as well as medical and economic aspects, should be pursued with vigour.

## 1. Introduction

A transurethral indwelling catheter (TUIC) is the most common form of urinary drainage perioperatively, during intensive care stays, or as short-term treatment of lower urinary tract dysfunction [1]. The TUIC should be removed when intensive care monitoring is completed, the bladder dysfunction has ceased, or when patients are mobile enough to empty their bladder independently [2]. Nevertheless, a significant proportion of patients suffering from acute severe neurological disorders, such as a stroke, experience prolonged periods of immobility and neurogenic lower urinary tract dysfunction (NLUTD) [3]. The symptoms of suprapontine and pontine lesions mainly consist of nocturia, urge incontinence, and increased urinary frequency because of neurogenic detrusor overactivity. While symptoms may resolve spontaneously in up to 71% of patients affected by a stroke, in several cases, they persist for a lifetime [3,4]. This causes significant burden on those affected and their caregivers, as the NLUTD leads to frequent, imperative micturition, and patients require significant assistance for micturition or changing pads [5]. Therefore, TUIC frequently persists until admission to rehabilitation or permanent care facilities, even though TUIC drainage is not a viable long-term solution. Potential complications include urethral damage, catheter-associated urinary tract infections (CAUTI) with avoidable antibiotic use, impaired quality of life, and further loss of bladder capacity [6,7]. Guidelines generally advise against the permanent use of TUIC, yet there is a paucity of guideline-based recommendations for dealing with urinary drainage in patients with severe neurological impairment [2]. Despite the need for systematic concepts for bladder management that provide recommendations on the timing, predictors, and contraindications of catheter removal [8,9], such concepts have not yet been developed [10]. Although there are some studies investigating health-related quality of life (HRQoL) regarding TUIC in lifelong indication [7,11,12,13], the impact of successful removal of the TUIC on the health-related well-being of those affected has not yet been published so far.

The aim of the study was therefore to prospectively investigate the impact of successful indwelling catheter removal on the health-related quality of life in patients with severe neurological impairment. These data should contribute to the development of a systematic concept for the bladder management in this special cohort.

## 2. Materials and Methods

### 2.1. Patients and Assessments

This monocentric, prospective, longitudinal study included 33 individuals who had been treated at a neurological rehabilitation centre between August 2020 and March 2023 due to the consequences of a severe acute neurological disease. A total of 183 individuals were considered, and 33 individuals were included according to the inclusion and exclusion criteria. Inclusion criteria were age > 18 years, wearing a TUIC, mental and verbal/nonverbal ability to answer questions and to indicate the desire to void, consent to participate in the study, independent transfer, if necessary, with the assistance of a caregiver, and medical indication for a TUIC removal. Exclusion criteria were faecal incontinence, acute urinary tract infections at the time of inclusion, pre-existing malignant urological conditions, and pre-existing NLUTD (e.g., in cases of spinal cord injury, Parkinson’s disease, or multiple sclerosis).

The participants were interviewed by two test administrators, who explained the procedure, the aim of the study, and obtained their consent to participate. On the first day of participation, age, gender, and the diagnosis underlying neurological rehabilitation were recorded. The Minimal Mental State Examination (MMSE) [14] and Dementia Detection Test (DemTec) [15] were performed to record the cognitive status of the patients. HRQoL was determined using the Short Form Health Survey 36 (SF-36) in the German version [16]. On the following day, the TUIC was removed and postvoid residual was measured by transabdominal ultrasound examination. Five days after the successful TUIC removal, the SF-36 was performed again, and urinary incontinence was recorded using the International Consultation on Incontinence Questionnaire—Urinary Incontinence Short Form (ICIQ-UI-SF) [17] in the German version [18]. Urinary incontinence was documented if the sum score was >0. The reasons for an unsuccessful TUIC removal were a post void residual > 150 mL and the inability to manage urinary incontinence with pads or condom urinal adequately.

The SF-36 is a general health questionnaire with eight subscales that can be assigned to two summary measures of subjective health. The subscales contain a varying number of questions. The Physical Component Summary measure (PCS) includes the scales Physical Functioning (PF) consisting of ten questions, Role Physical (RP) consisting of four questions, Bodily Pain (BP) consisting of two questions, and General Health (GH) consisting of five questions. The Mental Component Summary measure (MCS) comprises Vitality (VT) consisting of four questions, Social Functioning (SF) two questions, Role-Emotional (RE) with three questions and Mental Health (MH) with five questions. The SF 36 also contains two questions on general and current health status. The questions are to be answered using 3 to 6 item Likert scales and yes/no answers, which are converted into points using a scoring key. Average values for the eight dimensions are calculated from this [19,20]. Scores ranging from 0 to 100 are possible, with lower values indicating greater limitations in the respective subscale.

The Multiple Mini-Mental Status Examination (MMSE) [14] is designed to assess various cognitive functions. These functions include spatial orientation, memory, retention, attention, calculation, and language. A maximum of 30 points can be achieved. A score of 27 to 25 points indicates mild cognitive impairment, 24 to 21 points indicates mild dementia, below 20 points indicates moderate dementia, and below 10 points indicates severe dementia.

DemTect [15] is a short test that is independent of age and educational level and is used to detect MCI and dementia at an early stage. The maximum total score is 18 points. Cognitive performance corresponds to age at 13–18 points, 9–12 points indicate suspected mild cognitive impairment, and 8 points or less indicates suspected dementia. DemTect and MMSE are used to describe the cohort.

All participants or their legal representatives signed a written consent form to participate. Approval for the study was granted on 20 May 2020 by the Ethics Committee of the University Hospital Bonn (EK 118/20).

### 2.2. Statistical Analysis

Descriptive analysis and the comparison between the groups of successful vs. unsuccessful TUIC removal was performed using SPSS^®^, version 29.0 (IBM Corp., Armonk, NY, USA). The *t*-test for independent samples was used for DemTect and age, and the Mann–Whitney-U test was used for MMSE. All other analyses were performed using the R statistical programming language [21]. We assessed the differences of the mean values of the SF-36 subscales and the SF-36 summary measures before and after the TUIC removal using a one-sample *t*-test. Since we subtracted the initial from the final measurements, a negative value indicates a deterioration. The mean differences in SF-36 subscales and summary measurements were adjusted for age and gender using linear models. A *p*-value < 0.05 was considered statistically significant.

## 3. Results

### 3.1. Patients’ Characteristics and Results of MMST and DemTect

Our patient cohort consisted of *n*= 24 (73%) men and *n* = 9 (27%) women. The causes for neurological rehabilitation were stroke in *n* = 16 (48.5%), critical illness polyneuropathy in *n* = 8 (24.2%), intracranial haemorrhage/traumatic brain injury in *n* = 5 (15.1%), intracranial tumour in *n* = 2 (6.1%), and paresis of unknown origin in *n* = 2 (6.1%) cases. The mean age was 68.1 (SD 12.4) years (median 67, Q1 58, Q3 79; min 41, max 85).

In our cohort, *n* = 21 (63.6%) had a successful and *n* = 12 (36.4%) had an unsuccessful TUIC removal. In the group with successful TUIC removal, *n* = 10 (47.6%) had urinary incontinence and *n* = 11 (52.4%) patients were continent. Table 1 provides an overview of the descriptive statistics for the overall cohort and the sub cohorts with successful versus unsuccessful TUIC removal. These subcohorts had no statistically significant differences in the mean values of DemTec (mean diff. −0.226, T −0.159, *p* = 0.438), age (mean diff. −7.107, T −1.626, *p* = 0.057), and MMSE (U (*n*1 = 21; *n*2 = 12) =121; z = −0.189; *p* = 0.868).

### 3.2. Changes in the SF-36 Health-Related Quality of Life After TUIC Removal

To assess the HRQoL after a successful TUIC removal, we considered the differences between the initial and final measurements of the SF-36 subscales and summary measures. Since higher values in the SF-36 subscales represent a better quality of life, we chose the difference as final minus initial, so that positive values stand for an improvement and negative values stand for a deterioration in the HRQoL. Based on our cohort, we were able to demonstrate a significant improvement in the Physical Functioning and Role-Emotional subscales, as well as in the Mental Component Summary measure. Negative changes were observed in the Role-Physical and Bodily Pain subscales, and in the Physical Component Summary measure after TUIC removal. The latter did not show statistical significance. The values are summarised in Table 2.

**Figure 1:**
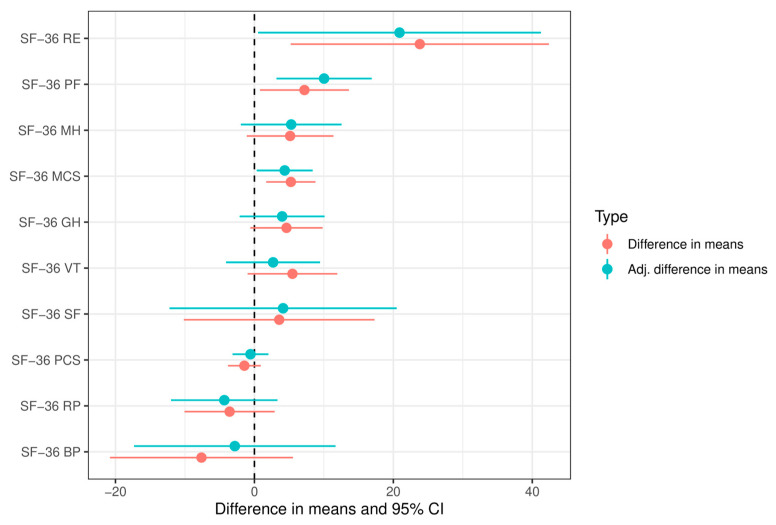
SF-36 subscales and component summary measures before and after the successful TUIC removal. Values less than zero indicate a deterioration. The confidence intervals are marked as lines. Statistical significance is present if the confidence interval does not intersect zero. PF Physical Functioning, RP Role-Physical, BP Bodily Pain, GH General Health, VT Vitality, SF Social Functioning, RE Role-Emotional, MH Mental Health, PCS Physical Component Summary, MCS Mental Component Summary.

Figure 1 depicts the changes in the SF-36 subscales and component summary measures with confidence intervals graphically.

### 3.3. Comparison of the SF-36 HRQoL Between Urinary Continent and Incontinent Patients

Initially, we compared the mean values of the SF-36 subscales and component summary measures of patients with and without urinary incontinence after the successful TUIC removal (SF-36_Post_). There were statistically significant differences in the Physical Component Summary measure, as well as in the subscales Bodily Pain and Physical Functioning. Continent patients had significantly higher values, which indicated a better quality of life. After adjusting for gender and age, a significant difference was shown for the Bodily Pain and Physical Component Summary measures.

A comparison of the longitudinal changes in SF-36 before and after the TUIC removal (Diff SF-36_post-pre_) between incontinent and continent patients showed a statistically significant difference in favour of continent patients for the Physical Functioning subscale. All other subscales, except Role-Emotional and General Health, show higher values for continent patients, but no statistical significance could be demonstrated for these subscales.

Table 3 and Table 4 provide a detailed overview of the comparison between incontinent and continent patients.

## 4. Discussion

The utilization of a TUIC should be based on clear medical indications [2,22]. Since transurethral urinary drainage can have many negative consequences for the patient, it is important to remove the TUIC as soon as possible or switch to other urinary drainage systems [23]. However, the reality in various medical care facilities is different [1]. Underlying reasons are complex. In addition to a shortage of nursing staff, there is a lack of structured guidelines for bladder management in people with severe, acute neurological disorders [1,10]. Neuro-urological expertise is becoming increasingly scarce, meaning that individual concepts based on urodynamic examination results were not developed on a consistent basis [24]. Thus, the TUIC transforms from an initially medically indicated device into the final bladder management concept, with the consequences of reduced bladder capacity, frequent urinary tract infections, and damaged urethras [6]. As bladder capacity is reduced, the viability of alternative procedures such as suprapubic urinary diversion, intermittent external catheterisation, or prompted voiding is decreased [25,26]. The self-determination and wishes of the affected individuals frequently become a secondary consideration [1].

Therefore, the aim of this study was to investigate the effect of successful TUIC removal on health-related quality of life and to contribute to the development of a structured bladder management for the cohort studied.

### 4.1. Main Findings

In our prospective study of 33 patients undergoing neurological rehabilitation, a longitudinal comparison of HRQoL before and after a successful TUIC removal showed statistically significant improvements in the SF-36 Physical Functioning and SF-36 Role-Emotional subscales as well as in the Mental Component Summary measure.

In a subgroup comparison of continent versus incontinent individuals after the TUIC removal, continent patients had better HRQoL in the SF-36_post_ Bodily Pain subscale and the Mental Component Summary measure. The longitudinal comparison showed a significantly higher increase in quality of life for continent patients in the SF-36 Physical Functioning subscale.

Therefore, our study showed evidence that the patient group examined had a better HRQoL after the successful TUIC removal. The urinary continent patients were the ones who benefited more from the TUIC removal in comparison.

### 4.2. Findings in the Context of Existing Evidence

The interactions between urinary catheters and quality of life have been investigated in several studies [7,11,12,13]. Their results showed that long-term urinary catheter use has a negative impact on the quality of life of those affected. In detail, concerns about catheter dysfunction and physical complaints are described [7,13]. We were able to show significant improvements in QoL in the SF-36 subscales of physical functioning, role emotional, and mental health after the TUIC removal. Christiaans et al. 2025 and Trautner et al. 2019 showed corresponding negative feelings among patients when the TUIC was worn permanently [7,13].

In contrast, a study on the effect of urinary catheters on QoL during acute postoperative hospital stays showed advantages of the catheter in terms of pain and patient comfort [27]. These results are reasonable with the focus on postoperative wound pain and mobilisation. Trautner et al. also highlighted the positive aspects of comfort provided by TUIC [7]. These results are consistent with our findings of a deterioration in bodily pain and role-physical after the TUIC removal because the pain caused by physical impairment due to repositioning or transfer becomes the main issue then. The patients must be moved more frequently for micturition or for incontinence care. The reduced bladder capacity and neurogenic detrusor overactivity after a stroke further exacerbate this situation. The decrease in HRQoL in role-physical (not being able to work as long as usual, being less productive, only being able to do certain things and having problems performing them) is also understandable in light of urgency symptoms or the increased effort required for bladder emptying [9].

The negative impact of urinary incontinence on QoL is well known and has been described in various postoperative and conservative settings [4,28,29,30]. Furthermore, this negative impact has also been demonstrated in various other studies specifically for post-stroke patients [8,9,31], and our study results are consistent with this phenomenon. Our continent patient group had significantly better post-TUIC removal scores in HRQoL for Physical Functioning and in the Physical Component Summary, as well as a significant increase in Physical Functioning in the before/after comparison. These findings are coherent with the pre-described impaired rehabilitation and the increased mortality in post-stroke patients with urinary incontinence [5,8,9,31] and emphasise the need for bladder management concepts. Therefore, our study results support the following implications for clinical practice.

### 4.3. Implications for Clinical Practice

The use of TUIC as a long-term concept should be avoided as far as possible. In addition to the frequently described negative consequences of TUIC care, such as CAUTI and urethral damage [6,32], our study indicates that catheter removal had a positive effect on the HRQoL of patients with acute severe neurological disease, especially when urinary continence was present. The cohort studied presents a particular challenge in terms of developing a urological care concept, as in addition to neurogenic dysfunction of the urinary tract, mobility, dexterity, cognition, and communication may also be impaired, exacerbating the negative effects of urinary incontinence [4,9]. Such neurological deficits can potentially result in the necessity for bladder management, even in cases where lower urinary tract function remains normal. These neurological deficits can even lead to the need for bladder management even when the lower urinary tract function is normal, i.e., where patients are unable to transfer independently for micturition [27].

There is a lack of suitable instruments for the development of a bladder management concept in the affected cohort that can reliably measure the effect of the medical procedure. The SF-36 is not suitable for assessing HRQoL in the cohort we are considering, as it contains many questions about life situations that the person no longer finds themselves in due to their illness [20]. This can lead to psychological stress for the respondent and unreliable results.

The limitations in the application of the SF-36 have also been reported in various other studies. For instance, the SF-36 was found to be incapable of adequately reflecting the incontinence-related HRQoL of female patients with meningomyelocele [33]. Another study investigated the influence of age on the results of the SF-36 in women with urinary incontinence and the age of the respondents led to differences in the results [34].

Although questionnaires on the quality of life of people with permanent indwelling catheters do exist, these only refer to necessary catheter long-term treatment and are focused on the process of catheter wearing [11,35].

The development of a questionnaire to evaluate the impact of urological symptoms specifically in individuals with severe neurological conditions is essential for the establishment and evaluation of treatment strategies for NLUTD in this specific group. For example, an adaption of the International Consultation on Incontinence Questionnaire Cognitively Impaired Elderly (ICIQ-Cog) would be desirable, which evaluates the HRQoL of those affected based on an external assessment and considers the burden on professional carers. The latter is also relevant in times of nursing staff shortages, increasing care needs among the population, and rising healthcare costs, and should be incorporated into future care concepts [36,37,38]. However, the current version of the ICIQ-Cog has not been definitively validated in the English language, and it specifically concerns the impact of urinary incontinence [39,40].

An additional approach could be the development of new devices for external urinary drainage. With the development of a “condom urinal for women”, the use of transurethral indwelling catheters due to mobility restrictions in women could be avoided. This could also be a solution for men who, for anatomical reasons, cannot use a conventional condom urinal reliably. The negative consequences of diaper use on infection, skin, and mental health could thus be avoided without invasive catheters. The amount of daily care required could be significantly reduced.

## 5. Conclusions

The investigated cohort showed an improvement in the psychological, emotional, and physical functioning aspects of the SF-36 HRQoL. Patients diagnosed with urinary incontinence exhibited a lower HRQoL when compared to the group of continent patients. There is a lack of suitable instruments for assessing quality of life in patients with acute severe neurological disorders. Consequently, developing appropriate questionnaires for diagnosis and therapy management should be a research priority, thereby contributing to the development of concepts for bladder management. Urinary incontinence should be treated as effectively as possible due to the numerous adverse effects it causes.

## 6. Limitations

Our study has some limitations, which will be addressed as follows. The main limitation is the small number of cases. The study was conducted during the peak of the COVID-19 pandemic, with numerous restrictions in place. The exceptional additional workload on nursing staff [41] and restrictions on access to clinics and patient rooms led to the low number of cases. This was a consequence of the additional nursing workload associated with TUIC removal and increased patient contact being avoided, as described in other studies [42]. The negative impact of the COVID-19 pandemic on clinical research has been described previously [43,44] and could not be influenced under the given circumstances. The low inclusion rate is also a reflection of the lack of a bladder management concept with structured guidelines for TUIC removal. Nevertheless, we would like to publish the present exploratory data, as the lack of data and low case numbers is a decisive factor in the failure of development process of these concepts [10].

Another limitation is the SF-36 Health Survey itself. A high share of patients was unable to answer the questions, some of which were complex and did not correspond to their current life situation. Furthermore, the SF-36 was not combined with a disease-specific questionnaire other than the ICIQ-SF UI. However, at present, there is no questionnaire that has been validated for the purpose of mapping HRQoL and the impact of TUIC removal. Particularly for a patient cohort with impaired communication skills, the possibility of a third-party assessment should be considered during the development of such a questionnaire.

The influence of non-neurogenic factors (bladder outlet obstruction due to prostatic hyperplasia or female genital descensus) on the successful TUIC removal was not considered in this study, as the study design was not intended for this purpose. This is a very important aspect for a systematic bladder management concept and must be included in future prospective analyses with a specially designed study protocol. Finally, we would like to mention that the urinary incontinence was not specified according to its severity or type of care (condom urinal, diapers). However, the size of the cohort did not allow for any appropriate subgroup analysis. This should be included into a future randomised, controlled study on the development of bladder management in patients suffering from acute severe neurological disorders.

## Figures and Tables

**Table 1:** Overview of the descriptive statistics for the overall cohort and the sub cohorts with successful versus unsuccessful TUIC removal.

Variable	Total	TUIC Removal	
		Unsuccessful	Successful
	*n* = 33	*n* = 12	*n* = 21
	Mean (SD)	Mean (SD)	Mean (SD)
	Min; Max	Min, Max	Min, Max
	Median (Q1, Q3)	Median (Q1, Q3)	Median (Q1, Q3)
Age	68.1 (12.4)	72.6 (8.9)	65.5 (13.5)
	41; 85	59; 85	41; 85
	67 (58.5, 79.0)	72.5 (65.1, 80.1)	62.0 (55.0, 78.0)
MMSE	24.6 (4.7)	24.3 (5.3)	24.8 (4.5)
	14.0, 30.0	14.0, 30.0	15.0, 30.0
	26.0 (21.0, 29.5)	25.0 (21.0, 29.8)	26.0 (21.5, 29.5)
DemTect	9.9 (3.9)	10.1 (3.8)	9.9 (4.1)
	1.0, 17.0	1.0, 14.0	4.0, 17.0
	10.0 (7.0, 13.0)	11.5 (7.5, 13.0)	10.0 (6.0, 14.0)
Gender	*n* (%)	*n*	*n*
	Male/female	Male/female	Male/female
	24 (73%)/9 (27%)	9 (75%)/3 (25%)	15 (71.4%)/6 (28.6%)

**Table 2:** Overview of the SF-36 subscales and component summary measures before and after the successful TUIC removal.

	TUIC Removal (*n* = 21)				
SF-36 Subscales	SF-36_Pre_	SF-36_Post_	Diff SF-36_Post-Pre_		
	Mean (SD)	Mean (SD)	Mean (SD)	Mean Diff.	Adjusted Mean Diff.
	Min, Max	Min, Max	Min, Max	95% CI	95% CI
	Median (Q1, Q3)	Median (Q1, Q3)	Median (Q1, Q3)	*p*-Value	*p*-Value
SF-36 PF	10.0 (14.0)	17.2 (22.7)	7.2 (14.3)	*7.2*	*10.0*
	0.0, 50.0	0.0, 70.0	−15.0, 55.0	*0.8*; *13.6*	*3.18*; *16.88*
	5.0 (0.0, 10.0)	5.0 (5.0, 15.0)	5.0 (0.0, 10.0)	*p* = *0.029*	*p* = *0.007*
SF-36 RP	10.7 (20.3)	7.1 (14.0)	−3.6 (22.8)	−3.57	−4.33
	0.0, 75.0	0.0, 50.0	−75.0, 25.0	−10.06; 2.91	−11.98; 3.32
	0.0 (0.0, 25.0)	0.0 (0.0, 0.0)	0.0 (0.0, 0.0)	*p* = 0.263	*p* = 0.249
SF-36 BP	62.8 (38.3)	55.1 (38.3)	−7.6 (30.7)	−7.62	2.82
	0.0, 100.0	0.0, 100.0	−100.0, 68.0	−20.79; 5.56	−17.31; 11.67
	62.0 (32.0, 100.0)	61.0 (22.0, 100.0)	0.0 (−20.0, 0.0)	*p* = 0.241	*p* = 0.687
SF-36 GH	41.0 (13.1)	45.6 (12.6)	4.6 (13.2)	4.62	3.99
	20.0, 62.0	25.0, 77.0	−20.0, 30.0	−0.58; 9.82	−2.1; 10.09
	40.0 (30.0, 50.0)	47.0 (35.0, 52.0)	2.0 (−3.0, 12.0)	*p* = 0.079	*p* = 0.185
SF-36 VT	44.5 (24.6)	50.0 (19.7)	5.5 (17.3)	5.48	2.69
	10.0, 95.0	20.0, 80.0	−25.0, 45.0	−0.98; 11.93	−4.06; 9.45
	45.0 (25.0, 60.0)	45.0 (40.0, 65.0)	5.0 (0.0, 15.0)	*p* = 0.092	*p* = 0.412
SF-36 SF	47.6 (35.7)	51.2 (38.1)	3.6 (31.4)	3.57	4.1
	0.0, 100.0	0.0, 100.0	−75.0, 50.0	−10.15; 17.3	−12.21; 20.47
	50.0 (12.5, 87.5)	62.5 (0.0, 87.5)	0.0 (0.0, 12.5)	*p* = 0.592	*p* = 0.601
SF-36 RE	46.0 (46.5)	69.8 (45.8)	23.8 (46.1)	*23.81*	*20.89*
	0.0, 100.0	0.0, 100.0	−66.7, 100.0	*5.23*; *42.39*	*0.54*; *41.24*
	33.3 (0.0, 100.0)	100.0 (0.0, 100.0)	0.0 (0.0, 66.7)	*p* = *0.015*	*p* = *0.045*
SF-36 MH	57.3 (29.2)	62.5 (25.3)	5.1 (15.3)	5.14	5.3
	4.0, 100.0	12.0, 96.0	−16.0, 36.0	−1.1; 11.38	−1.95; 12.54
	56.0 (36.0, 76.0)	72.0 (48.0, 76.0)	8.0 (−8.0, 16.0)	*p* = 0.101	*p* = 0.141
SF-36 PCS	27.4 (6.7)	25.9 (6.0)	−1.4 (6.1)	−1.44	−0.57
	15.5, 40.0	17.5, 37.2	−15.6, 8.4	−3.81; 0.92	−3.14; 2.01
	26.0 (22.5, 31.4)	25.3 (21.5, 29.8)	0.7 (−3.2, 2.0)	*p* = 0.216	*p* = 0.649
SF-36 MCS	45.3 (17.7)	50.5 (14.2)	5.2 (9.5)	*5.25*	*4.36*
	17.9, 76.1	20.2, 68.9	−7.2, 35.4	*1.7*; *8.8*	*0.34*; *8.38*
	44.8 (29.5, 60.1)	52.1 (41.5, 60.5)	4.7 (−2.0, 10.6)	*p* = *0.006*	*p* = *0.035*

SF-36_Post_ final measurement, SF-36_Pre_ initial measurement, PF Physical Functioning, RP Role-Physical, BP Bodily Pain, GH General Health, VT Vitality, SF Social Functioning, RE Role-Emotional, MH Mental Health, PCS Physical Component Summary, MCS Mental Component Summary. Statistically significant results are shown in italics.

**Table 3:** Comparison of the SF-36_post_ subscales and component summary measures of continent and incontinent patients after successful TUIC removal.

SF-36 Subscale	SF-36_Post_ Mean (SD) Min, Max Median (Q1, Q3) Continent (*n* = 11)	SF-36_Post_ Mean (SD) Min, Max Median (Q1, Q3) Incontinent (*n* = 10)	Mean Diff. 95% CI *p*-Value	Adjusted Mean Diff. 95% CI *p*-Value
SF-36 PF	27.3 (27.8)	6.1 (5.2)	*−21.15*	−15.97
	0.0, 70.0	0.0, 15.0	*(−39.98; −2.31)*	(−36.82; 4.87)
	10.0 (5.0, 60.0)	5.0 (5.0, 6.3)	*p* = *0.031*	*p* = 0.124
SF-36 RP	6.8 (16.2)	7.5 (12.1)	0.68	0.08
	0.0, 50.0	0.0, 25.0	(−12.31; 13.67)	(−15.08; 15.24)
	0.0 (0.0, 0.0)	0.0 (0.0, 25.0)	*p* = 0.914	*p* = 0.991
SF-36 BP	69.7 (34.5)	39.1 (37.3)	−30.63	*−42.7*
	0.0, 100.0	0.0, 100.0	(−63.62; 2.36)	*(−77.34*; *−8.06)*
	72.0 (41.0, 100.0)	26.5 (12.0, 64.0)	*p* = 0.067	*p* = *0.019*
SF-36 GH	45.5 (14.6)	45.7 (10.7)	0.15	−0.85
	25.0, 77.04	30.0, 60.0	(−11.48; 11.79)	(−14.41; 12.71)
	7.0 (32.0, 52.0)	48.5 (35.0, 55.0)	*p* = 0.978	*p* = 0.896
SF-36 VT	54.1 (22.5)	45.5 (16.2)	−8.59	−10.31
	20.0, 80.0	20.0, 70.0	(−26.43; 9.25)	(−29.56; 8.94)
	45.0 (35.0, 80.0)	42.5 (40.0, 60.0)	*p* = 0.325	*p* = 0.274
SF-36 SF	51.1 (38.1)	51.3 (40.2)	0.11	1.31
	0.0, 100.0	0.0, 100.0	(−35.79; 36.01)	(−39.83; 42.46)
	62.5 (0.0, 87.5)	56.3 (0.0, 87.5)	*p* = 0.995	*p* = 0.947
SF-36 RE	72.7 (46.7)	66.7 (47.1)	−6.06	−3.46
	0.0, 100.0	0.0, 100.0	(−49.02; 36.9)	(−51.47; 0 44.55)
	100.0 (0.0, 100.0)	100.0 (0.0, 100.0)	*p* = 0.771	*p* = 0.881
SF-36 MH	62.2 (24.2)	62.8 (27.8)	0.62	−8.14
	12.0, 96.0	12.0, 96.0	(−23.39; 24.63)	(−33.48; 17.2)
	64.0 (48.0, 76.0)	72.0 (36.0, 84.0)	*p* = 0.957	*p* = 0.507
SF-36 PCS	29.7 (5.22)	21.9 (3.72)	*−7.8*	*−7.76*
	21.7, 37.2	17.5, 28.1	*(−11.92*; *−3.67)*	*(−12.47*; *−3.05)*
	29.8 (25.3, 33.9)	21.2 (18.5, 24.9)	*p* = *0.001*	*p* = *0.003*
SF-36 MCS	49.7 (15.09)	51.5 (13.87)	1.78	−0.28
	20.2, 68.9	27.4, 68.9	(−11.45; 15.01)	(−14.97; 14.4)
	52.1 (41.5, 60.5)	54.7 (38.3, 62.0)	*p* = 0.782	*p* = 0.968

SF-36_Post_ final measurement, PF Physical Functioning, RP Role-Physical, BP Bodily Pain, GH General Health, VT Vitality, SF Social Functioning, RE Role-Emotional, MH Mental Health, PCS Physical Component Summary, MCS Mental Component Summary. Statistically significant results are shown in italics.

**Table 4:** Comparison of the longitudinal changes of the SF-36 subscales and component summary measures before and after the successful TUIC removal between continent and incontinent patients.

SF-36 Subscale	Continent (*n* = 11) Diff SF-36_Post-Pre_ Mean (SD) Min, Max Median (Q1, Q3)	Incontinent (*n* = 10) Diff SF-36_Post-Pre_ Mean (SD) Min, Max Median (Q1, Q3)	Mean Diff. 95% CI *p*-Value	Adjusted Mean Diff. 95% CI *p*-Value
SF-36 PF	13.2 (16.7)	0.6 (6.95)	*−12.56*	−11.36
	0.0, 55.0	−15.0, 10.0	*(−24.42*; *−0.7)*	(−24.42; 1.7)
	5.0 (0.0, 20.0)	0.0 (0.0, 5.0)	*p* = *0.04*	*p* = 0.084
SF-36 RP	−4.5 (15.08)	−2.5 (29.93)	2.05	4.4
	−25.0, 25.0	−75.0, 25.0	(−20.64; 24.73)	(−20.16; 28.96)
	0.0 (−25.0, 0.0)	0.0 (0.0, 25.0)	*p* = 0.849	*p* = 0.71
SF-36 BP	−3.1 (27.34)	−12.6 (34.8)	−9.51	−11.78
	−39.0, 68.0	−100.0, 21.0	(−38.51; 19.49)	(−41.92; 18.37)
	0.0 (−22.0, 0.0)	0.0 (−20.0, 2.0)	*p* = 0.49	*p* = 0.421
SF-36 GH	3.4 (13.54)	6.0 (13.5)	2.64	5.28
	−20.0, 22.0	−10.0, 30.0	(−9.74; 15.01)	(−8.71; 19.28)
	2.0 (−3.0, 17.0)	5.0 (−5.0, 10.0)	*p* = 0.66	*p* = 0.437
SF-36 VT	7.7 (18.08)	3.0 (17.03)	−4.73	−2.16
	−15.0, 45.0	−25.0, 30.0	(−20.77; 11.31)	(−19.85; 15.52)
	0.0 (0.0, 15.0)	7.5 (−10.0, 15.0)	*p* = 0.545	*p* = 0.799
SF-36 SF	8 (37.2)	−1.3 (24.62)	−9.2	−11.25
	−75.0, 50.0	−62.5, 37.5	(−37.95; 19.54)	(−44.8; 22.3)
	12.5 (−12.5, 50.0)	0.0 (0.0, 0.0)	*p* = 0.509	*p* = 0.489
SF-36 RE	39.4 (46.71)	6.7 (41.0)	−32.73	−18.5
	0.0, 100.0	−66.7, 100.0	(−72.79; 7.33)	(−61.93; 24.86)
	0.0 (0.0, 100.0)	0.0 (0.0, 0.0)	*p* = 0.104	*p* = 0.38
SF-36 MH	7.6 (17.93)	2.4 (12.25)	−5.24	−6.22
	−16.0, 36.0	−16.0, 20.0	(−19.22; 8.75)	(−22.64; 10.2)
	8.0 (−8.0, 20.0)	4.0 (−8.0, 12.0)	*p* = 0.441	*p* = 0.435
SF-36 PCS	−1.2 (6.9)	−1.7 (5.43)	−0.56	−0.74
	−14.3, 8.4	−15.6, 2.9	(−6.22; 5.09)	(−6.81; 5.33)
	0.7 (−8.1, 2.5)	−0.7 (−3.2, 1.8)	*p* = 0.837	*p* = 0.801
SF-36 MCS	8.0 (11.08)	2.2 (6.71)	−5.79	−4.28
	−6.0, 35.46.3	−7.2, 14.2	(−14.15; 2.57)	(−13.92; 5.35)
	(0.3, 13.6)	1.8 (−2.8, 4.7)	*p* = 0.162	*p* = 0.361

SF-36_Post_ final measurement, SF-36_Pre_ initial measurement, PF Physical Functioning, RP Role-Physical, BP Bodily Pain, GH General Health, VT Vitality, SF Social Functioning, RE Role-Emotional, MH Mental Health, PCS Physical Component Summary, MCS Mental Component Summary). Statistically significant results are shown in italics.

## Data Availability

The original contributions presented in this study are incorporated within the article. Should further inquiries be required, they should be directed to the corresponding authors.

## Abbreviations

The following abbreviations are used in this manuscript:

BP	Bodily Pain
CAUTI	Catheter Associated Urinary Tract Infection
COVID-19	Corona Virus Disease 2019
GH	General Health
HRQoL	Health Related Quality of Life
ICIQ-SF UI	International Continence Society Questionnaire
ICIQ Cog	International Consultation on Incontinence Questionnaire Cognitively Impaired Elderly
MCS	Mental Component Summary
MH	Mental Health
NLUTD	Neurogenic Lower Urinary Tract Dysfunction
PCS	Physical Component Summary
PF	Physical Functioning
RE	Role-Emotional
RP	Role-Physical
SF	Social Functioning
SF-36	Short Form 36 Health Survey
TUIC	Transurethral Indwelling Catheter
VT	Vitality
